# The role of dopamine decline, astrocyte reactivity, and cerebral small-vessel disease in cognitive aging

**DOI:** 10.1177/0271678X261441065

**Published:** 2026-04-11

**Authors:** Vanessa Crine, Jarkko Johansson, Olivia Ericsson, Anders Wåhlin, Jan Axelsson, Micael Anderssson, Lars Nyberg, Nina Karalija

**Affiliations:** 1 Department of Medical and Translational Biology, Umeå University, Umeå, Sweden; 2 Umeå Center for Functional Brain Imaging (UFBI), Umeå University, Umeå, Sweden; 3 Department of Diagnostics and Intervention, Umeå University, Umeå, Sweden; 4 Department of Applied Physics and Electronics, Umeå University, Umeå, Sweden

**Keywords:** ^11^C-L-deprenyl-D_2_, ^18^F-FE-PE2I, positron emission tomography, cerebral small-vessel disease, cognition

## Abstract

Several aging-related brain changes have been associated with unsuccessful cognitive aging, including dopamine decline, increased astrocyte reactivity, and cerebral small-vessel disease (SVD). We hypothesized that dopamine decline is exacerbated in older adults with higher measures of astrocyte reactivity and cerebral SVD, and that reduced dopamine integrity would be the strongest predictor of lower cognitive performance. Healthy adults (*n* = 55, ages: 60–79 years) underwent positron emission tomography with ligands ^18^F-FE-PE2I to estimate levels of dopamine transporters (DAT) and ^11^C-L-deprenyl-D_2_ to estimate levels of monoamine oxidase B (MAO-B)—a protein expressed to some degree by neurons but mainly by astrocytes. Cerebral SVD was assessed by white matter lesion volumes from magnetic resonance images. General cognition was evaluated via tests of episodic memory, working memory, and perceptual speed. Contrary to expectations, increased MAO-B levels (indicative of astrocyte reactivity) were associated with higher DAT availability (*r* = 0.53, *p* < 0.001) and reduced white matter lesion volumes (*r* = −0.33, *p* = 0.021). Reduced DAT availability was more strongly related to reduced MAO-B (*r* = 0.47, *p* < 0.001) than white matter lesion volumes (*r* = −0.22, *p* > 0.05), and only DAT was a significant predictor of cognition (*r* = 0.36, *p* = 0.032). These findings underscore the critical role of dopamine for cognition and indicate reduced glial function to underlie dopaminergic losses.

## Introduction

Normal aging is associated with decline in several cognitive functions, albeit with large individual differences that have been related to brain maintenance.^1^ It is well-established that the brain undergoes structural, functional, vascular, and neurochemical changes. Still, we lack knowledge of which of these brain changes that are most critical for cognitive functions at older ages.^2,3^ Here, we present a unique brain imaging study that maps three brain processes that have each been associated with aging-related cognitive decline and imminent dementia: dopamine (DA) decline, astrocyte reactivity, and cerebral small-vessel disease (SVD).^4–6^ Importantly, these indicators have so far not been evaluated within the same cohort, and consequently, their interrelations and individual and shared importance for cognitive aging remain inconclusive.

The DA system undergoes significant pre- and postsynaptic reductions across the lifespan,^7–9^ with accentuated losses in groups with dementia.^4^ Losses of DA receptors and DA transporters (DAT) have been suggested to underlie cognitive malfunction in normal aging as well as in disorders.^10–12^ Pioneering pharmacological studies showed that augmentation of DA signaling can alleviate memory impairments^13^—a finding that has been replicated in human studies.^14,15^ Since then, numerous cross-sectional human brain imaging studies employing positron emission tomography (PET) have shown that striatal DA receptors and DAT availability predict between-person differences in several age-sensitive cognitive functions, including episodic memory, working memory, executive functions, and perceptual speed.^16–20^ Recently, this link has been validated with longitudinal data, showing that decline in DA D2-like receptors and general cognition is correlated in older adults.^21^

One important knowledge gap is whether DA decline serves as a stronger brain correlate of cognitive performance, compared to other co-occurring brain changes. Chronic low-grade inflammation is recognized as a hallmark of normal aging and stems from aging-related immune system changes.^22,23^ Long-term increase of systemic inflammatory markers can trigger neuroinflammation which impairs the milieu within the brain, with concomitant decline in brain function and cognition.^24^ It has been suggested that inflammation serves as one factor behind late-life DA decline and dysfunction of DA-related behaviors.^25^ We have provided tentative evidence that elevated systemic inflammation is associated with reduced striatal DA D2-like receptor availability, but this link was only found in older men.^26^ Here, we extend these investigations to one indicator of inflammatory processes in the brain: astrocyte reactivity. During neuroinflammation, the most abundant glial cells in the brain, astrocytes, adopt a reactive phenotype upon which they alter their gene expression, morphology, and function.^27^ Reactive astrocytes upregulate their expression of the protein monoamine oxidase-B (MAO-B),^28,29^ and consequently, astrocyte reactivity can be evaluated with radioligands that bind selectively to MAO-B (e.g. ^11^C-L-deprenyl-D_2_) and PET.^30^ MAO-B is also expressed by serotonergic neurons^29,31^; hence part of the ligand binding likely represents this fraction. ^11^C-L-deprenyl-D_2_ binding increases with advancing age^32,33^ and further elevations are observed in several neurodegenerative disorders.^34^

Cerebral SVD is characterized by damage to the smaller vessels of the brain primarily caused by atherosclerosis, cerebral amyloid angiopathy, and hypertension.^6,35^ The pathophysiology of SVD involve demyelination, ischemia, atrophy, and inflammation.^6,36^ Manifestations of cerebral SVD (e.g. white matter lesions, lacunes, microbleeds, perivascular space dilation) can be evaluated via magnetic resonance imaging (MRI) and are highly prevalent in older adults.^37^ Meta-analyses converge on that higher severity of white matter lesions is associated with decline in general cognition and increased risk of forthcoming dementia.^38–40^ Cognitive deficits may arise in direct response to the pathophysiology of SVD,^36^ or possibly, due to compromised neurotransmission in regions proximal to SVD manifestations.^41^ White matter lesions manifest around the basal ganglia and has been associated with reduced striatal DA D1- and D2-like receptors and DAT.^8,41,42^

Taken together, inflammation and vascular dysfunction may serve as two factors that modulate DA system integrity in aging, but also in some vascular and neurogenerative disorders (e.g. stroke and Parkinson’s disease).^43–45^ Currently, there is lack of an integrative approach that tests associations among these processes. The research questions of this work were: (i) Do astrocyte reactivity and SVD contribute to individual difference in DA integrity at older ages? (ii) Does DA decline hold a more critical role for cognitive performance than SVD and astrocyte reactivity? and (iii) Are there apparent sex differences in these brain and cognitive markers? To answer these questions, 55 healthy older adults underwent PET with ligands ^11^C-L-deprenyl-D_2_ (^11^C-DED) and ^18^F-FE-PE2I (^18^F-PE2I) for assessment of MAO-B levels and striatal DAT availability, MRI to estimate two marker of cerebral SVD (white matter lesions and lacunes), and tests of episodic memory, working memory, and perceptual speed. The specific hypotheses were:

1. DAT availability, astrocyte reactivity, and SVD are part of one detrimental cascade where elevated astrocyte reactivity, larger white matter lesions, and lacunes impairs the DA system (i.e. are negatively correlated with striatal DAT availability).^42,46,47^
2. Given the well-established DA-cognition link in aging,^11^ we hypothesized that striatal DAT availability would be the strongest predictor of general cognition. That is, we excepted DAT availability to be positively correlated with all three cognitive domains.
3. Based on our previous work, we expected men to demonstrate lower striatal DAT availability, larger white matter lesion volumes, and higher astrocyte reactivity.^26,48^

## Subjects and methods

This study was approved by the Swedish Ethical Review Authority (registration number: 2022-01586-01) and was conducted in accordance with the Declaration of Helsinki. All participants provided signed written informed consent prior to any testing.

### Participants

Enrollment to this cross-sectional study took place between years 2020 and 2023. Individuals between ages 60 and 80 years were randomly selected from the population registry of Umeå, a city in northern Sweden. They were offered participation via a letter that provided a concise overview of the study’s objectives, design, and duration. Exclusion criteria concerned conditions and medical treatments that affect brain functioning and cognition. Specifically, these included diabetes, ongoing treatment for cancer, Parkinson’s disease, dementia or other neurological disorders, psychiatric disorders, previous head trauma or stroke, mental and physical disability. Additional exclusion factors concerned conditions that could affect test performance (severe visual or auditory impairment, limited proficiency in Swedish) or obstruct brain imaging (claustrophobia, anticipated difficulties in remaining still during a 1-h period, metal implants). Furthermore, participants performed a Mini Mental State Examination (MMSE) at the beginning of the first session, where a minimum of 26 points (of maximum 30) served as an inclusion criterion for study enrollment.

The sample size was guided by a priori statistical power analyses, showing that a sample size between 55 and 70 individuals is needed for 80% power to detect a medium effect size (Cohen’s *f*^2^ = 0.15 and *p* = 0.05) in a multiple linear regression model of *R*^2^ change with one to three tested predictors and at least one covariate (age is always adjusted for). The final sample consisted of 55 healthy older adults between ages 60 and 79 years (27 men, 28 women; mean age: 67.4 ± 5.1; Table 1). Data collection was distributed over 2 separate days and was completed within 2 weeks for most participants (mean time between sessions: 4.3 days, standard deviation: 3.9 days), but longer for 11 participants (mean time between sessions: 29.7 days, standard deviation: 13.9 days). During the first day, participants reported lifestyle and health-related data, performed the first part of the cognitive test battery (MMSE and working memory tasks) and underwent two PET sessions with ^11^C-DED, and then, ^18^F-PE2I. At the second day of data collection, participants performed the second part of the cognitive test battery (episodic memory and perceptual speed tasks) and underwent MRI.

**Table 1.** Sample descriptives. Frequencies, or mean values and SDs are presented for lifestyle and health-related variables.

Lifestyle and health	Frequency (%) or mean ± SD
Sex	27 men, 28 women (49% vs 51%)
Age	67.4 ± 5.1
MMSE	29.0 ± 1.1
Occupational status	
Working	43%
Retired	57%
Education (years)	14.6 ± 3.4
Hypertension diagnosis	55%
Systolic bp (mmHg)	143 ± 14.5
Diastolic bp (mmHg)	83.6 ± 8.8
Pulse	70.9 ± 11.4
BMI	27.4 ± 5.5
Nicotine consumption	
Smoking	4%
Snus	20%

MMSE: Mini Mental State Examination; bp: blood pressure; SD: standard deviations.

### Brain imaging

MRI was performed using a 3T GE Discovery MR 750 scanner (General Electric, Milwaukee, WI, USA) equipped with a 32-channel phased-array head coil for the first 10 participants. Following a major scanner upgrade, the remaining 45 individuals underwent MRI with a 3T GE SIGNA Premier system equipped with a 48-channel phased-array head coil. The same PET scanner was used for the whole sample (Discovery PET/CT 690; General Electric, Milwaukee, WI, USA). All 55 participants underwent MRI and ^11^C-DED/PET. ^18^F-PE2I/PET data was, however, missing for four participants due to unwillingness to undergo another PET session or due to technical issues.

#### Regional volumes

High resolution anatomical T1-weighted images were acquired by a 3D fast spoiled gradient-echo (FSPGR) sequence at the older scanner system, and for a few that were scanned with the upgraded scanner system (*n* = 16 in total). For most scanned with the newer scanner system (*n* = 39), T1-weighted images were acquired via a magnetization prepared-rapid gradient echo (MPRAGE) sequence. Parameters for the FSPGR sequence at the 3T GE Discovery MR 750 scanner were: 176 axial slices, thickness = 1 mm, repetition time (TR) = 8.2 ms, echo-time (TE) = 3.2 ms, flip angle: 12°, inversion time (TI): 450 ms, field of view (FOV): 25 × 20 cm with resolution 0.98 mm (isotropic) up-sampled to 0.49 mm.

Parameters for the FSPGR sequence at the 3T GE SIGNA Premier system were 180 axial slices, thickness = 1 mm, repetition time (TR) = 6.8 ms, echo-time (TE) = 2.9 ms, flip angle: 12°, inversion time (TI): 450 ms, field of view (FOV): 25 × 25 cm with resolution 0.98 mm (isotropic) up-sampled to 0.49 mm. Parameters for the MPRAGE sequence at the 3T GE SIGNA Premier system were 336 sagittal slices, thickness = 0.5 mm, TR = 2500 ms, TE = 2800 ms, flip angle: 8°, TI: 1000 ms, FOV: 24 × 24 cm with resolution 0.98 mm (isotropic) up-sampled to 0.47 mm.

Estimates of gray matter, white matter, and lateral ventricle size were derived from T1 images using the FreeSurfer software, version 7.4.1. (https://surfer.nmr.mgh.harvard.edu).^49^ Quality of segmentations was visually inspected (by authors VC and MA) and considered adequate. Subcortical and cortical gray-matter segmentations were used to define regions of interest (ROIs) for ^18^F-PE2I and ^11^C-DED. Volumetric differences can influence ligand binding estimations, however, we found no differences for volumes estimated with FSPGR versus MPRAGE sequences for the target or reference regions of PET analyses (*striatum*: (*t*(54) = −1.7, *p* = 0.092), *hippocampus*: (*t*(54) = 1.7, *p* = 0.104), *thalamus*: (*t*(54) = 1.3, *p* = 0.207), *cortex*: (*t*(54) = 1.7, *p* = 0.100), *cerebellum*: (*t*(54) = 0.7, *p* = 0.457)).

#### Dopamine transporter availability and astrocyte reactivity

Participants underwent two PET scans during the same day to estimate MAO-B levels via the uptake rate constant (*Ki_ref_*) for ^11^C-DED, and then, to estimate DAT availability via ^18^F-PE2I binding potential (BP_ND_). PET sessions were separated by 3–4 h (<0.2% of ^11^C-DED remaining when the ^18^F-PE2I started, i.e. levels that are not detectable in the ^18^F-PE2I scan). Each scan started with a low-dose CT scan (20 mA, 120 kV, 0.8 s/revolution) for attenuation-correction purposes. Fifty-five-min dynamic PET scans were acquired during resting conditions following a bolus infusion of 321.4 ± 24.1 MBq ^11^C-DED or 144.1 ± 10.0 MBq ^18^F-PE2I. Number of time frames were 26 for ^11^C-DED/PET (9 × 20, 6 × 60, 5 × 120 och 6 × 360 s) and 18 for ^18^F-PE2I/PET (9 × 120 + 3 × 180 + 3 × 260 + 3 × 300 s). The same reconstruction methods were used for both scans. Specifically, attenuation- and decay-corrected images (47 slices, field of view = 25 cm, 256 × 256-pixel transaxial images, voxel size = 0.977 × 0.977 × 3.27 mm^3^) were reconstructed with the iterative VUE Point HD-SharpIR algorithm (GE). Reconstruction parameters were six iterations, 24 subsets, 3.0 mm post filtering, yielding FWHM of 3.2 mm.^50^

All pre-processing of PET images was performed with the Statistical Parametric Mapping software (SPM12). For PET images of both tracers, images were motion-corrected by aligning frames to a single reference frame (here frame 10—chosen for its stability and representative signal quality) and then, an average was made from all corrected frames and frame-alignment was repeated once more. Next, a mean image was created from the third to the last frame (excluding the initial two frames to minimize noise) and co-registered with the T1-weighted images. FreeSurfer-derived segmentation of T1 images were employed to define ROIs. Reference tissue modeling was performed for both tracers in ROIs. Time (*t*) versus activity (*C*) curves were recorded for each region of interest and the reference region (cerebellum). Due to different binding characteristics for the two tracers (DED binds irreversibly while FE-PE2I binding is reversible), two different established models were employed. ^18^F-PE2I BP_ND_ was estimated with the multilinear reference tissue model using all time frames.^51 ^11^^C-DED *Ki_ref_* was calculated with a semi-quantitative reference-Patlak method^52^ using time frames 4–26 (i.e. in the steady-state phase, allowing for linear fitting). In the Patlak model, the reference was calculated as *C_ref_(t)* = *C_cer_(t)·e*^−0.04 t^, where *C_cer_(t)* is the time-activity for cerebellum. This method has been established to achieve *Ki_ref_* values that correlate well with in vitro deprenyl binding and PET estimates adjusted for metabolite-corrected plasma input function.^52,53^

**Figure 1.**
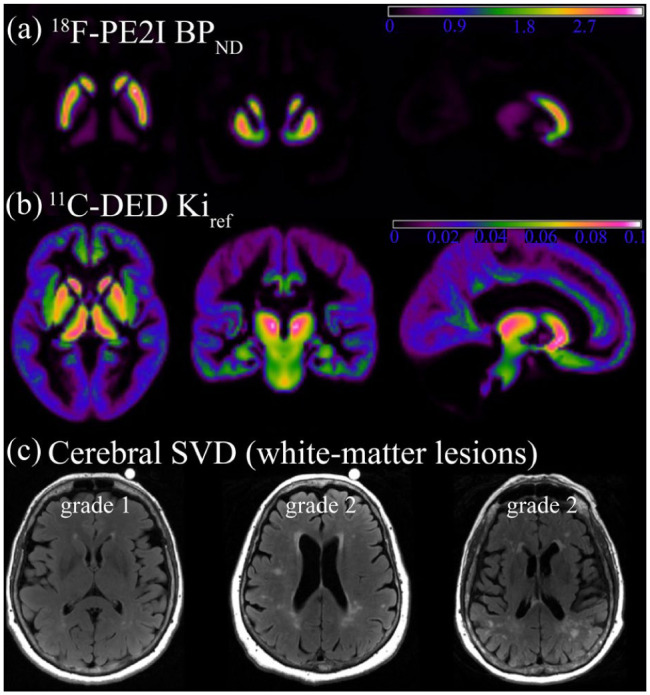
In vivo imaging of dopamine transporters and astrocyte reactivity with PET, and cerebral small-vessel disease (white matter lesions) with magnetic resonance imaging: (a) group mean images of striatal DAT availability via 18F-PE2I BP_ND_, (b) group mean images of astrocyte reactivity estimated via ¹¹C-DED *Ki_ref_*, and (c) axial FLAIR images with white matter lesions of severity grade 1 (mild) and grade 2 (moderate) according to the Fazekas scale. PET: positron emission tomography.

Mean ^18^F-PE2I and ^11^C-DED images are shown in Figure 1, and mean values are found in Table 2. In addition, we complemented ROI-based estimations with voxelwise analyses of ^11^C-DED uptake. For the latter, ^11^C-DED PET images were normalized to an average group template and aligned into stereotactic space of the Montreal Neurological Institute (smoothing: 8.0 mm full width at half maximum gaussian filter).

**Table 2.** Mean values for striatal DAT availability (via ^18^F-PE2I BP_ND_), astrocyte reactivity (via ^11^C-DED *Ki_ref_*), and cerebral small-vessel disease (white matter lesions).

Brain measures	Mean ± SD (frequency, %)
DAT availability (^18^F-PE2I BP_ND_)	
Caudate	2.24 ± 0.52
Putamen	3.31 ± 0.59
Astrocyte reactivity (^11^C-DED *Ki_ref_*)	
Caudate	0.06 ± 0.01
Putamen	0.07 ± 0.00
Thalamus	0.06 ± 0.00
Hippocampus	0.05 ± 0.00
Cortex	0.04 ± 0.00
Cerebral SVD (white matter lesions)	
Volume (ml)	2.0 ± 2.9
Lesion severity grade:	
0: None	1 (2%)
1: Mild	38 (69%)
2: Moderate	16 (29%)
3: Severe	0

SD: standard deviation; BP_ND_: binding potential; *Ki_ref_*: uptake rate constant; DAT: dopamine transporter.

#### Cerebral small-vessel disease

T1 and FLAIR images were assessed for presence of SVD indicators, and specifically, white matter lesions and lacunar infarcts. White matter lesions were identified as hyperintensities in FLAIR images (48 axial slices, thickness: 3 mm). Parameter for FLAIR images were: TR = 8000 ms, TI: 2250 ms, flip angle: 111°, field of view: 24 × 21.6 cm, and in-plane resolution = 0.94 × 0.94 mm, up-sampled to 0.469 × 0.469 mm. TE was 125 ms at the older system and 121 ms at the new system. Subsequently, FLAIR images were co-registered to T1-weighted images and white matter lesions were automatically segmented using the LST-AI algorithm from the LST toolbox.^54^ To improve specificity, we used FreeSurfer-derived white matter segmentations, ensuring that only lesions within white matter were identified. All lesion maps were visually inspected. Lesion volumes were then calculated by converting voxel counts to lesion volumes (ml). White matter lesion volumes were relatively modest for the sample (2.0 ± 2.9 ml), but with large individual differences (range for lesion volumes: 0.1–14.7 ml). Of these, the maximum value (14.7 ml) was excluded, as it was a statistical outlier (>3.3 standard deviations from the mean). The number of subjects with lesion data was thus *n* = 54.

In addition, lesion severity was visually inspected and graded according to the Fazekas scale.^55^ Lesions in periventricular regions and in deep white matter were graded as follows: 0 = no lesions, 1 = thin lining around ventricles or punctual foci in deep white matter, 2 = beginning confluence, 3 = large, confluent lesions. Only one participant had a grade of 0, whereas most participants had lesion severity grades of 1 or 2. No participants had a grade of 3 (Table 2). Total lesion volumes for these groups were 0.8 ± 0.8 ml for Fazekas grade 1, 4.9 ± 4.0 ml for grade 2 (*p* < 0.001). Examples of mild versus moderate lesions severity are shown in Figure 1(c).

Lacunes manifest as small (3–15 mm) dark circular cavities in T1 and FLAIR images, sometimes with hyperintense edges in the latter.^37^ Only two individuals had lacunes in the present sample, and therefore, this marker was not included in the statistical analyses. That is, white matter lesions served as a proxy of SVD in the current work.

#### Perfusion

3D pseudo-continuous arterial spin labeling was acquired with background suppression and spiral readout. Following parameters were used during a 5-min image acquisition (at both scanner systems). Labeling time: 1.5 s, post-labeling delay time: 1.5 s, field of view: 24 cm, slice thickness: 4 mm, acquisition resolution: 8 × 512 (arms × data points), number of averages: 3. Reconstructed voxel size was 1.875 × 1.875 × 4 mm^3^. Quantitative cerebral blood flow maps were aligned to each participant’s T1 image to extract mean perfusion values in FreeSurfer-defined gray matter regions (computed as the average of the individual perfusion estimates weighted by volume). Perfusion is expressed in ml/100 g/min. Two participants had missing perfusion data (*n* = 53).

### Cognitive assessment

The main cognitive test battery consisted of computerized tests of episodic memory, working memory, and perceptual speed. The cognitive test battery has been used in another aging study (see detailed description in reference 56). In brief, each of the three domains were assessed via three separate tasks using letter-, number-, and figure-based data, respectively. All tests were repeated and preceded by a practice trial. Before each task, short written instructions were read by participants and discussed with the test instructor. Test trials were preceded by practice trials. Episodic memory was assessed via word recall (maximum score = 32), number-word recall (maximum score = 16), and object-position recall (maximum score = 24; Spearman–Brown coefficient = 0.65, 0.58, and 0.77, respectively). Working memory was assessed with a letter-updating task (maximum score = 48), a columnized numerical 3-back task (maximum score = 108), and a spatial-updating task (maximum score = 30; Cronbach’s alpha = 0.68, 0.91, and 0.65, respectively). Perceptual speed was assessed via letter-, number-, and figure-comparison tasks (Spearman–Brown coefficients = 0.96, 0.95, and 0.93, respectively). Participants were instructed to respond as fast and as accurately as possible. Performance in the speed tasks was calculated as the number of correct responses divided by the total response time (in ms), multiplied by 60,000, that is, correct responses per minute. As in the previous study,^56^ test scores were normally distributed for all nine tests and without ceiling effects (see Figure S1). Low test scores were, however, observed for one episodic memory test (number-word recall), as was also the case in the previous study.

Subsequently, scores were summarized across trials of the nine tests and standardized to *z*-scores. An average *z*-score was calculated per ability (episodic memory, working memory, perceptual speed), which were then averaged to form one composite score of general cognition. Upon missing data for any of the individual cognitive tests, *z*-scores per cognitive ability were calculated from the remaining tests. Note that the first 10 participants only performed episodic memory tests. Furthermore, one participant only underwent the working memory sessions. Consequently, the effective sample for the cognitive analyses were *n* = 54 for episodic memory, *n* = 45 for working memory, *n* = 44 for perceptual speed and for general cognition.

### Health and lifestyle assessments

Participants provided information about their medical history, prescribed medications, nicotine consumption, and some demographic factors (e.g. educational attainment and employment status). Select physical parameters were measured, including weight and height to calculate BMI, blood pressure, and pulse. As is seen in Table 1, half of the sample had hypertension, most had a BMI over 25 (overweight), however few consumed nicotine.

### Statistical analysis

All data analyses were conducted using the R software (version 4.2.2) and R Studio (version: 2023.06.0 + 421).^57,58^ Univariate outliers were defined as values ±3.29 standard deviations from the mean^59^ and excluded as pairwise deletions (*n* = 1 for perceptual speed and lesions, see their respective sections above). No significant multivariate outliers were identified (Mahalanobis distance and *p* threshold of 0.001). Number of observations ranged between *n* = 44 and 55 for the brain and cognitive variables, which were all normally distributed (*brain:* −0.25 to 0.31 for skewness and −0.82 to 1.09 for kurtosis; *cognition*: −0.14 to 0.58 for skewness and −0.75 to 0.01 for kurtosis; see Figure S1). The distribution of white matter lesion volumes was positively skewed, and therefore, lesion volumes were log-transformed (skewness: 0.30 and kurtosis: −0.74 following transformation). Descriptive data are presented with mean or sum values, standard deviations, or frequencies. Zero-order correlations were conducted to assess associations among brain variables, cognition, and demographic or lifestyle-related variables. Partial correlations were used to adjust for effects of, for example, age for brain–brain and brain–cognition associations. Correlations are reported with Pearson’s correlation coefficient (*r*). The significance level for all tests was 5%, adjusted for multiple comparison using Bonferroni correction.

Previous research has highlighted the importance of striatal DA markers for cognitive aging (see e.g., Bäckman et al.,^16^ Rieckmann et al.,^18^ and Nyberg et al.^60^). Hence, the striatum (caudate and putamen) was the a priori ROI for DAT estimation. In accordance with previous work,^61 ^18^^F-PE2I BP_ND_ was highest in the striatum (Figure 1(a)) and correlated between caudate and putamen (*r* = 0.78, *p* < 0.001). Significant, but lower, correlations were found for ^18^F-PE2I binding in thalamus and the brainstem (Table S1). DAT availability was averaged for caudate and putamen and standardized into a composite (*Z*) score. A principal component analysis (PCA) was performed to identify the correlative structure of MAO-B binding (^11^C-DED *Ki_ref_*) across the cortex, hippocampus, and thalamus. DA neurons do not express MAO-B.^29^ Still, we excluded the striatum from the PCA to avoid potential circularity, as the striatum has high DAT levels as well as MAO-B expression.^62,63^ The PCA revealed that a first component accounted for 78% of the variance (see factor loadings in Table S2). The first factor had an eigenvalue of 2.3 whereas eigenvalues were <1 for the other components. Correlations for ^11^C-DED uptake in cortex and subcortical regions are presented in Table S3. Following, factor scores of the first component of ^11^C-DED uptake represented astrocytic reactivity in the statistical analyses. As mentioned earlier, general cognition was represented by an average *z*-score for the three cognitive domains, and SVD by log-transformed values for total white matter lesion volumes.

Between-group comparisons were assessed via *t*-tests or analysis of covariance (ANCOVA) for continuous variables, and with chi-square test for categorical variables. Multiple linear regression models were employed to investigate associations among brain variables (Table 3) and their associations to general cognition (Table 4). Specifically, in the models presented in Table 3, we estimated the individual contributions of white matter lesion volumes and astrocyte reactivity for variance in DAT availability. In the models presented in Table 4, we tested whether astrocyte reactivity and white matter lesions explained further variance in cognition as compared to DAT availability alone. Model comparisons were estimated via *R*^2^ change. Age was a covariate in both models.

**Table 3.** Brain correlates of DAT availability. Results are reported for multiple regression models of striatal DAT. Covariates age and sex were entered in the first model, cerebral small-vessel disease (white matter lesion volumes) in the second model, and astrocyte reactivity in the third model.

Regression models	Variable	β	*t*	*r*	*p*
Model 1					
*F* = 5.1, *p* = 0.009	Age	−0.27	−2.0	−0.28	**0.048**
Adjusted *R*^2^ = 0.15	Sex	−0.38	−2.8	−0.38	**0.007**
Model 2					
*F* = 5.8, *p* = 0.002	White matter lesions	−0.35	−2.5	−0.34	**0.018**
Adjusted *R*^2^ = 0.23	Age	−0.12	−0.8	−0.12	0.418
	Sex	−0.31	−2.4	−0.33	**0.022**
Model 3					
*F* = 8.7, *p* < 0.001	Astrocyte reactivity	0.45	3.6	0.47	**<0.001**
Adjusted *R*^2^ = 0.39	White matter lesions	−0.20	−1.5	−0.22	0.143
	Age	−0.32	−2.3	−0.32	**0.027**
	Sex	−0.29	−2.5	−0.35	**0.016**

DAT: dopamine transporter; *r*: Pearson’s correlation coefficient for partial correlations.

Significant associations are marked with bold font.

**Table 4.** Brain correlates of general cognition. Results are reported for multiple regression models of general cognition. The covariate age was entered in the first model, and DAT availability in the second model. The third model also included cerebral small-vessel disease (white matter lesion volumes) and astrocyte reactivity.

Regression models	Variable	β	*t*	*r*	*p*
Model 1					
*F* = 9.0, *p* = 0.005	Age	−0.44	−3.0	−0.44	**0.005**
Adjusted *R*^2^ = 0.17					
Model 2					
*F* = 9.2, *p* < 0.001	DAT availability	0.39	2.8	0.42	**0.009**
Adjusted *R*^2^ = 0.30	Age	−0.35	−2.5	−0.38	**0.018**
Model 3					
*F* = 4.4, *p* = 0.006	DAT availability	0.41	2.2	0.36	**0.032**
Adjusted *R*^2^ = 0.26	Lesion volume	−0.01	−0.1	−0.01	0.958
	Astrocyte reactivity	−0.06	−0.3	−0.05	0.758
	Age	−0.31	−1.7	−0.28	0.096

DAT: dopamine transporter; *r*: Pearson’s correlation coefficient for partial correlations.

Significant associations are marked with bold font.

## Results

### Associations among DAT availability, astrocyte reactivity, and white matter lesions

Striatal DAT availability (^18^F-PE2I BP_ND_; Figure 1(a)) showed an expected negative, albeit non-significant, association with age (*r* = −0.20, *p* = 0.153), and was higher in men than in women (*z* scores: 0.34 ± 0.95 vs −0.28 ± 0.92; *t*(49) = 2.4, *p* = 0.022). Astrocyte reactivity (^11^C-DED *Ki_ref_*) was highest in the striatum and thalamus, but also sizeable in the hippocampus and the cortex (Figure 1(b) and Table 2). Correlations of ^11^C-DED *Ki_ref_* across brain regions were moderate to strong, with *r* values ranging from 0.56 to 0.83 (Table S3). Older age was positively associated with astrocyte reactivity (*r* = 0.36, *p* = 0.008), and no apparent differences were found between men and women (*t*(53) = 1.3, *p* = 0.212). White matter lesion volumes (Figure 1(c)) were relatively modest for this sample, with no cases presenting with severe lesions (Table 2). Lesion volumes increased with advancing age (*r* = 0.42, *p* = 0.001) and were similar for men and women (*t*(52) = 0.7, *p* = 0.465).

Partial correlations, with adjustment for age, showed that white matter lesion volumes were negatively associated with striatal DAT availability (*r* = −0.39, *p* = 0.006). Unexpectedly, astrocyte reactivity was negatively associated with white matter lesion volumes (*r* = −0.33, *p* = 0.021) and positively associated with striatal DAT availability (*r* = 0.53, *p* < 0.001). That is, higher astrocyte reactivity was found in those with lower white matter lesion burden and higher DAT availability. We also present correlations between ^18^F-PE2I BP_ND_ and ^11^C-DED *Ki_ref_* across several ROIs (Table S4) and from a voxelwise analysis where average striatal ^18^F-PE2I BP_ND_ was regressed onto a whole-brain ^11^C-DED *Ki_ref_* map, with age and sex as covariates (Figure S2). These analyses showed wide-spread positive correlations between ^18^F-PE2I BP_ND_ and ^11^C-DED, but no negative associations (threshold for the exploratory voxelwise analysis was *p* < 0.001, uncorrected). Previous work showed that atrophy, cerebral blood flow, and BMI affect ligand uptake.^64–66^ Here, all three factors were correlated with ^18^F-PE2I binding (BMI: *r* = −0.53, total gray matter volume: *r* = 0.37, mean gray matter perfusion: *r* = 0.37, all *p* < 0.01), while only BMI was significantly associated with ^11^C-DED uptake (*r* = −0.43, *p* = 0.001; *r* values: 0.08 and 0.12 for total gray matter volume and perfusion). The correlation between ^18^F-PE2I BP_ND_ and ^11^C-DED *Ki_ref_* survived, however, adjustment for BMI in addition to age (*r* = 0.40, *p* = 0.004).

**Figure 2.**
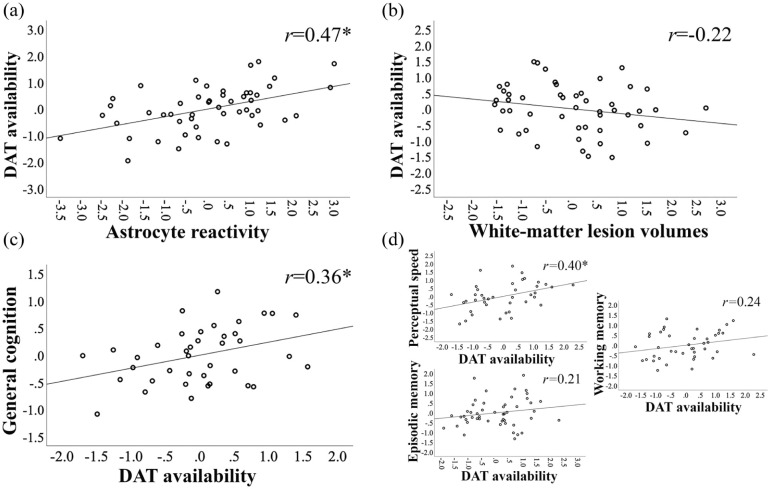
Associations among DAT availability, astrocyte reactivity, cerebral small-vessel disease (white matter lesions), and cognition. Partial correlation plots are shown for (a) astrocyte reactivity (^11^C-DED *Ki_ref_*) versus striatal DAT availability (^18^F-PE2I BP_ND_), (b) white matter lesion volume versus DAT availability, and (c) DAT availability versus general cognition. These plots represent the main results of the third models of Tables 3 (a, b) and 4 (c). Partial correlations, adjusted for age, are also shown for striatal DAT availability versus the separate cognitive domains (d). Correlations are reported with Pearson’s correlation coefficient (*r*). DAT: dopamine transporter. *p < 0.05.

Multiple regression models were conducted to examine the independent associations, and shared effects, of lesion volumes and astrocyte reactivity in relation to DAT availability (Table 3). White matter lesion volumes explained significant variance in DAT when modeled together with covariates age and sex (23% explained variance in model 2, *F*(1,46) = 6.0 and *p* = 0.018 for *R*^2^ change between models 1 and 2). The addition of astrocyte reactivity to a third model explained significantly more variance in DAT availability (39% explained variance; *F*(1,45) = 13.0 and *p* < 0.001 for *R*^2^ change between models 2 and 3), however, the association with lesion volumes was reduced and no longer significant. Partial plots for associations of the third model are shown in Figure 2(a) and (b). As a post-hoc analysis, we further adjusted for hypertension (use of antihypertensive agents according to medication lists), as hypertension has been related with DA integrity previously^47^ and is a principal risk factor of SVD.^35^ A model with independent variables: astrocyte reactivity (β = 0.52, *p* < 0.001), white-matter lesions volumes (β = −0.28, *p* = 0.033), hypertension (β = −0.59, *p* = 0.011), and age (β = −0.24, *p* = 0.082) explained 40% of variance in DAT availability (*F* = 9.0, *p* < 0.001). Hence, adjusting for hypertension did not change the outcome, but shows that hypertension (even if medicated) is associated with lower DA integrity.

### Brain correlates of general cognition

Older age was associated with poorer cognitive performance (*r* = −0.50, *p* < 0.001). Cognitive performance did not differ between men and women (*t*(42) = 0.5, *p* = 0.612) and was not significantly correlated with BMI (*r* = −0.05, *p* = 0.726), educational attainment (*r* = 0.29, *p* = 0.057), or perfusion (*r* = 0.28, *p* = 0.051). In support of the link between DA and cognition in aging (hypothesis 2), a linear regression analysis showed that DAT availability was positively associated with cognitive performance, beyond the effects of age (*F*(1,36) = 7.7 and *p* = 0.009 for *R*^2^ change between models 1 and 2; Table 4). Together, DAT availability and age explained 30% of variance in general cognition. White matter lesion volumes and astrocyte reactivity were not associated with cognitive performance (*F*(2,34) = 0.5 and *p* = 0.952 for *R*^2^ change between models 2 and 3). A partial plot for the association between DAT availability and general cognition (from the third model of Table 4) is shown in Figure 2(c). In a post-hoc analysis, we further adjusted for hypertension. A model with independent variables: DAT availability, hypertension, and age explained 26% of variance in cognition (*F* = 9.0, *p* < 0.001). In this model, DAT availability (β = 0.18, *p* = 0.033) and age (β = −0.25, *p* = 0.003) were significantly associated with cognition, but not hypertension (β = −0.18, *p* = 0.258). While the focus in the present work is on general cognition, we also present partial correlations between DAT availability and each separate cognitive domain, while adjusting for age (Figure 2(d)). All three abilities showed positive associations to DAT availability; however, strongest (and significant) associations were found with perceptual speed.

## Discussion

Previous work have advocated cerebral SVD and neuroinflammation as factors modulating DA integrity in normal aging^8,25^ and disease,^44,45^ however, comprehensive tests of this triad are currently missing. Here, we investigated the notion that DA decline, dysfunction to cerebral vasculature, and astrocyte reactivity are part of one detrimental cascade of events in the aging brain that underlies aging-related cognitive decline. We replicated previous associations between white-matter lesions and DAT availability,^42^ while showing that reduced astrocyte reactivity serves as a stronger brain correlate of reduced DA integrity than white matter lesions. In support of the correlative triad among aging, DA, and cognition,^11^ we identified a significant association between DAT availability and general cognition. Importantly, we show that DA decline is a stronger predictor of cognition than white matter lesions and astrocyte reactivity. In fact, white matter lesions and astrocyte reactivity were not significantly associated with cognition, which is surprising as meta-analytic evidence shows that higher SVD severity and elevated burden of pro-inflammatory markers are found in mild cognitive impairment and dementia.^40,67^ Furthermore, elevated astrocyte reactivity is observed in the early stages of dementia pathology, when mild cognitive impairment starts to manifest.^68^ Collectively, these data support a pivotal role of striatal DA for cognitive aging, and thus, underscores the importance of sparing the nigrostriatal DA system. Possibly, DA decline constitutes a risk factor of severe cognitive decline, as DA reductions are further exacerbated in dementia.^4^ The mesocortical and mesolimbic DA pathways are also critical for cognitive processes (see e.g., work by Goldman-Rakic^69^ and Lisman et al.^70^), but were not studied here due to low DAT expression in extrastriatal regions.

Associations among DA decline, SVD, and astrocyte reactivity has, to the best of our knowledge, not been studied within the same cohort previously. Earlier work has, however, shown pair-wise associations between white matter lesions and several DA markers, including D1- and D2-like receptors^8,41,71^ and DAT.^42^ In recent work, we further showed that burden of confluent white matter lesions and lacunes severity forecast the degree of prospective DA receptor decline within the striatum, where those with only minimal lesions and no lacunes are spared from aging-related DA receptor losses.^47^ SVD is a risk factor of imminent stroke,^38^ which in turn is also associated with DA reductions.^43^ This points towards ischemia, oxidative stress, and inflammation, that is, events associated with SVD and strokes, hampering the DA system. One novel finding here is that astrocyte reactivity is a stronger factor than white matter lesions in predicting individual differences in DA integrity. Astrocytes are involved in many physiological functions and serve to uphold homeostasis within the brain. To exemplify, astrocytes regulate levels of various molecules in nervous tissue, governs synaptic plasticity, and is a critical constituent of the blood–brain barrier and the glymphatic system.^27,72^ MAO-B is primarily expressed in astrocytes, as demonstrated via immunohistochemistry and autoradiography in postmortem human brain tissue.^29,31,73^ Excessive astrocyte reactivity is found in Parkinson’s disease and suggested as one mechanism behind DA decline.^74^ In support of this view, induction of elevated MAO-B expression in astrocytes resulted in Parkinson-like DA cell loss in mice.^46^ Another role of MAO-B in glial cells is to metabolize DA in the human brain, and thus, regulate DA levels.^63^ The relationship between astrocytes and DA neurons is reciprocal, as DA in turn shapes astrocytic signaling and their inflammatory response.^75^ Increased activation of DA D2-like receptors on astrocytes was found to suppress inflammation, and in turn, the vulnerability of DA neurons to neurotoxic agents.^76^

Astrocytes upregulate their MAO-B expression along the course of normal aging, which is interpreted as astrocytes becoming more reactive.^33,77^ A positive association was found between ^11^C-DED uptake and age also in the current work. MAO-B levels are further exacerbated in several neurodegenerative disorders,^34^ including the early stages of Alzheimer’s disease.^68,78^ Therefore, our a priori hypothesis states that higher MAO-B levels represent more reactive, and potentially neurotoxic, astrocytes that are expected to accompany increased severity of SVD and DA reductions. Instead, the present findings show that higher astrocyte reactivity is associated with *higher* brain maintenance, including higher DAT availability and lower lesion volumes. This unexpected finding may relate to the heterogeneity of astrocytes, being neuroprotective in healthy contexts and neurotoxic in pathological conditions.^27^ That is, the MAO-B values observed in our healthy sample may represent healthy (non-reactive) astrocytes that serve to support brain function,^78^ including DA integrity. Average global *Ki_ref_* values extracted with the same method in a younger sample, albeit including healthy controls and cases with multiple sclerosis, ranged approximately between 0.03 and 0.05 (see Table 2 in Hedman et al.^53^). As expected, our older sample had slightly higher average *Ki_ref_* values (between 0.050 and 0.065). Still, we cannot make conclusions of the functional state of the astrocytes studied in our work, as that would require further molecular characterization.^27^ It should be further noted that MAO-B is also expressed by serotonergic neurons,^29,31^ hence we cannot rule out that the DAT-MAO-B relationship may, at least in part, represent DA-serotonin associations.

DAT availability was intercorrelated with white matter lesions and astrocyte reactivity, and thus possibly part of the same neurobiological cascade in aging. Importantly, only DAT availability was significantly associated with general cognitive performance, beyond the effects of age. Positive associations were found between DAT and all cognitive abilities, but highest correlations were found with perceptual speed. Similarly, we recently reported that decline in DA D2-like receptor availability is associated with reductions in general cognition, but most strongly with perceptual speed.^21^ Mechanisms by which striatal DA control general cognition may include influencing prefrontal processing via striato-thalamo-cortical loops^79,80^ and modulation of signal-to-noise ratios at synapses, where higher DA levels are associated with better information processing.^81^ The present sample had a relatively low burden of white matter lesions and none of the participants presented with severe lesions or lacunes. It is possible that when SVD, but also astrocyte reactivity, reaches detrimental levels, these processes also contribute to cognitive malfunction.^40,82^ In healthy samples with relatively low lesion volumes, lesions are mainly located in white matter tracts that are proximal to the striatum (corpus callosum and corona radiata).^41^ The proximity to lesions (i.e. areas with ischemia, oxidative stress, and inflammation) may impair the striatal milieu and lead to DA losses. In samples with higher prevalence of confluent lesions, it is motivated to assess whether disruption of specific white matter tracts is more detrimental to some cognitive functions.

The cerebellum was used as the reference region for the graphical modeling of DAT and MAO-B levels. While the cerebellum is largely devoid of DAT,^83^ cerebellar ^11^C-DED binding is significantly lower, but not silent, as compared to other regions.^84^ Arterial blood sampling would thus have allowed for more accurate modeling of ^11^C-DED binding.^53^ That said, the graphical approach employed here yielded estimates that correlate well with in vitro binding of deprenyl as well as with in vivo estimations of ^11^C-DED uptake with plasma input function.^52,53^ We further found that the rank order among regional binding was coherent with earlier reports for healthy samples, with the highest binding of ^11^C-DED seen in the basal ganglia, thalamus, followed by hippocampus, and cortical gray matter regions.^77,85^ The regional differences do not indicate that astrocytes are more reactive in some regions, but rather reflect astrocyte heterogeneity across the brain.^86^ Lower values can also stem from underestimated *Ki_ref_* values in cortex, due to partial volume effects and noise sensitivity in low uptake region.^77^ Despite regional difference,^11^C-DED uptake was highly correlated across brain regions.

The sample size of this study is larger than most in vivo DA studies^7^ and was determined via a priori power analyses. Given that the effect sizes were larger than anticipated (models explained about 40% of variance in DAT availability and 25% of cognitive differences), post hoc power analyses show that we have about 95% power for the analyses presented here. Still, we do not have sufficient power to detect weaker main effects that may exist, for example, between lesions or astrocyte reactivity and cognition in this healthy sample, or weaker between-group differences for men and women (see hypothesis 3). Previous work has suggested that older men have elevated SVD severity and inflammation levels, and reduced levels of DA markers.^26,48,87^ Here, men and women showed comparable levels of white matter lesions and astrocyte reactivity, however, striatal DAT availability was higher in men than in women. Lastly, the cross-sectional design of this study prevents us from establishing causal relationships between SVD, astrocyte reactivity, DA function, and cognitive decline over time. Longitudinal studies are thus needed to clarify these dynamics.

## Conclusion

This study integrated multiple neurobiological markers to advance our understanding of aging-related cognitive decline. By simultaneously assessing DAT availability, astrocyte reactivity, and white matter lesions within the same cohort, the current study offers a framework to explore the interplay between these processes. While each of these brain processes have been suggested to modulate cognitive aging trajectories, findings from the present study highlight DA integrity as the strongest predictor of cognitive performance at older ages. Future studies should test the generalizability of the findings by assessing these relationships with other DA and astrocytic markers (e.g. D1-like or D2-like receptors, glial fibrillary acidic protein).

## Supplemental Material

sj-docx-1-jcb-10.1177_0271678X261441065 – Supplemental material for The role of dopamine decline, astrocyte reactivity, and cerebral small-vessel disease in cognitive aging Supplemental material, sj-docx-1-jcb-10.1177_0271678X261441065 for The role of dopamine decline, astrocyte reactivity, and cerebral small-vessel disease in cognitive aging by Vanessa Crine, Jarkko Johansson, Olivia Ericsson, Anders Wåhlin, Jan Axelsson, Micael Anderssson, Lars Nyberg and Nina Karalija in Journal of Cerebral Blood Flow & Metabolism
