# Supplementary material for: The role of dopamine decline, astrocyte reactivity, and cerebral small-vessel disease in cognitive aging
Source: J Cereb Blood Flow Metab. 2026 Apr 11:0271678X261441065. Online ahead of print. doi: 10.1177/0271678X261441065 (PMC13070112; doi:10.1177/0271678X261441065)
Supplement: sj-docx-1-jcb-10.1177_0271678X261441065 – Supplemental material for The role of dopamine decline, astrocyte reactivity, and cerebral small-vessel disease in cognitive aging [file sj-docx-1-jcb-10.1177_0271678X261441065.docx]

Figure S1. Histograms for general cognitive performance (A), calculated as the average of episodic memory (B), working memory (C), and perceptual speed (D). These display the distributions of standardized (z) scores. Raw test scores are shown for the three separate tests of each cognitive domain.

**
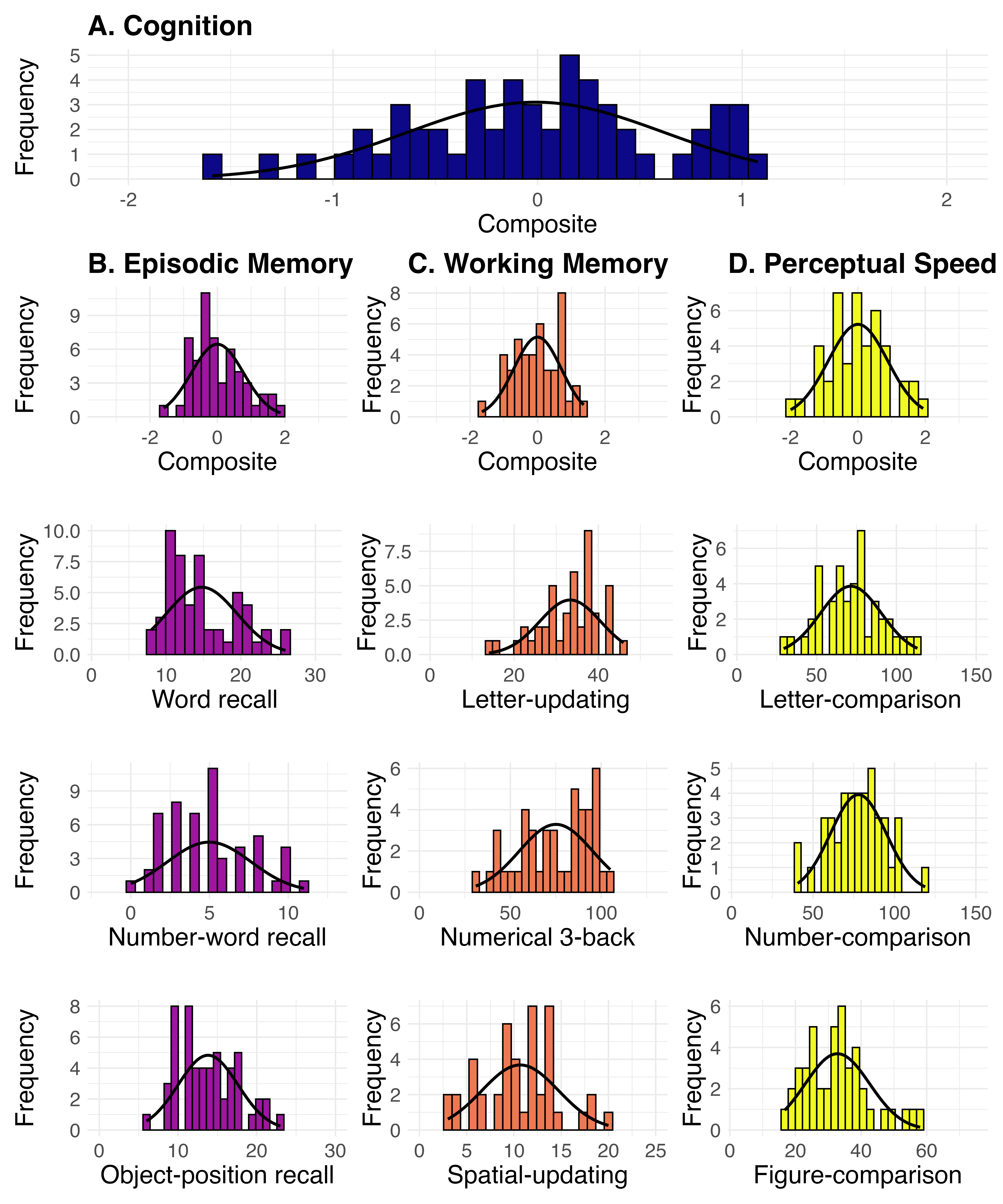
**

Figure S2. Correlation between striatal DA transporter availability (via straital^18^F-PE2I BP_ND_ in the striatal ROI) and voxelwise astrocyte reactivity (^11^C-DED *Ki_ref_*). Brain regions showing a positive correlation are displayed at an uncorrected voxel-level threshold of *p*<0.001 (with adjustment for age and sex). The color scale represents t-values.


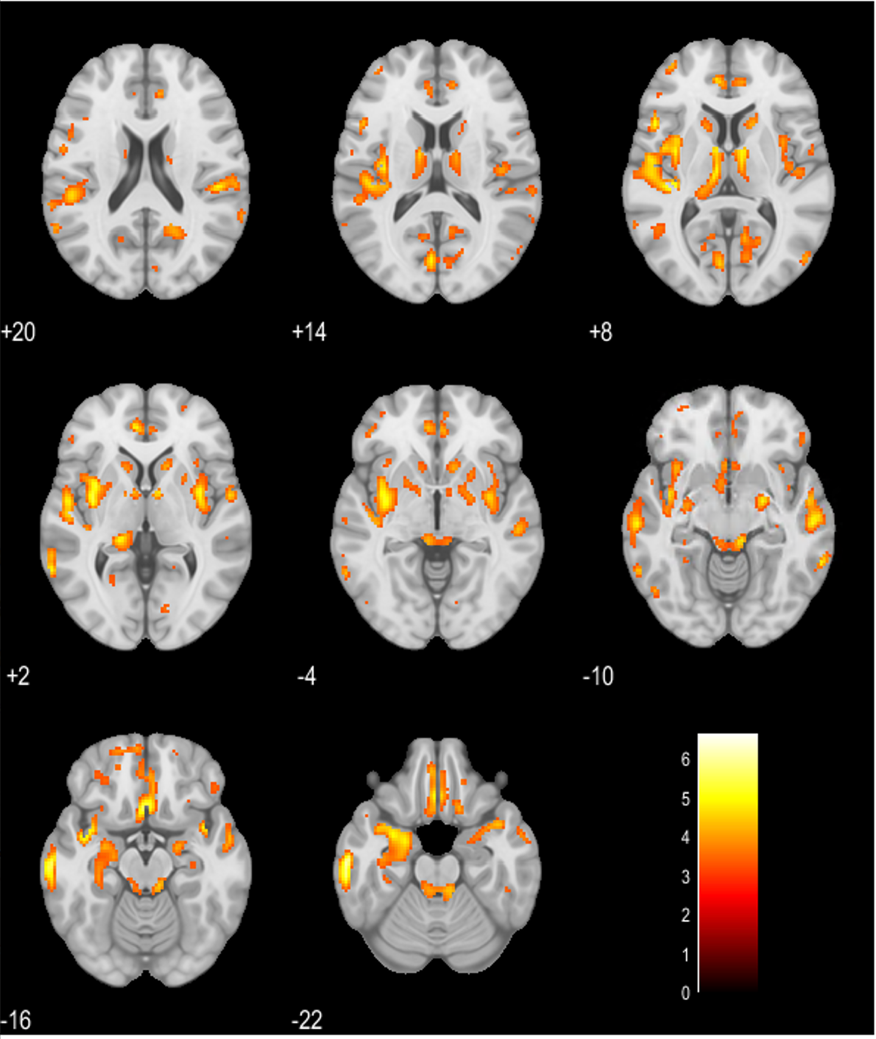


Table S1. Correlations among regional DA transporter (DAT) availability (i.e., ^18^F-PE2I BP_ND_ values). Zero-order correlations are shown above the diagonal (in black font), and partial correlations adjusted for age are found below the diagonal (in blue font).

|  | **Caudate** | **Putamen** | **Thalamus** | **Brainstem** |
| --- | --- | --- | --- | --- |
| **Caudate** |  | 0.78 | 0.59 | 0.32 |
| **Putamen** | 0.77 |  | 0.44 | 0.34 |
| **Thalamus** | 0.64 | 0.46 |  | 0.67 |
| **Brainstem** | 0.41 | 0.41 | 0.67 |  |

Note: All correlations are statistically significant (*p*<0.05).

Table S2. Results of the principal component analysis for ^11^C-DED *Ki_ref_* values across cortex, hippocampus, and thalamus. Explained variance and factor loadings per brain region are reported.

|  | **First principal component** |
| --- | --- |
| Explained variance  Eigenvalue  Thalamus  Hippocampus  Cortex | 78%  2.33  0.57  0.55  0.60 |

Table S3. Correlations among regional ^11^C-DED *Ki_ref_* values. Zero-order correlations are shown above the diagonal (in black font), and partial correlations adjusted for age are found below the diagonal (in blue font).

|  | **Caudate** | **Putamen** | **Thalamus** | **Cortex** | **Hippocampus** |
| --- | --- | --- | --- | --- | --- |
| **Caudate** |  | 0.64 | 0.61 | 0.56 | 0.65 |
| **Putamen** | 0.69 |  | 0.77 | 0.83 | 0.71 |
| **Thalamus** | 0.67 | 0.70 |  | 0.74 | 0.57 |
| **Cortex** | 0.57 | 0.80 | 0.69 |  | 0.68 |
| **Hippocampus** | 0.64 | 0.77 | 0.63 | 0.71 |  |

Note: All correlations are statistically significant (*p*<0.001).

Table S4. Correlations among regional ^18^F-PE2I BP_ND_ and ^11^C-DED *Ki_ref_* values. Zero-order correlations are shown in black font and partial correlations adjusted for age are found in parentheses (in blue font).

| **^18^F-PE2I BP_ND_** | | | | |
| --- | --- | --- | --- | --- |
|  | Caudate | Putamen | Thalamus | Brainstem |
| **^11^C-DED Ki_ref_** | | | | |
| Caudate | 0.59* (0.63*) | 0.32* (0.34*) | 0.45* (0.44*) | 0.21 (0.19) |
| Putamen | 0.26 (0.43*) | 0.31* (0.45*) | 0.32* (0.30*) | 0.38* (0.29*) |
| Thalamus | 0.23 (0.42*) | 0.24 (0.38*) | 0.33* (0.31*) | 0.39* (0.30*) |
| Hippocampus | 0.49* (0.52*) | 0.39* (0.40*) | 0.37* (0.37*) | 0.35* (0.35*) |
| Cortex | 0.34* (0.47*) | 0.37* (0.47*) | 0.50* (0.50*) | 0.51* (0.45*) |

Note: statistically significant correlations (*p*<0.05) are highlighted with an asterisk.
